# Off-putting! No red, no ripe: methylglyoxal inhibits fruit ripening

**DOI:** 10.1093/plphys/kiad239

**Published:** 2023-04-19

**Authors:** Divya Mishra

**Affiliations:** Assistant Features Editor, Plant Physiology, American Society of Plant Biologists, USA; Department of Botany, University of Wisconsin, Madison, WI 53706, USA

Eating ripe fruit is pleasurable. Ripened fruits have flavor and texture with substantial nutritional values. Fruit ripening is a highly coordinated process of various physiological, biochemical, molecular, and genetic changes that convert a fruit from unripe (green) to a ripened (colored) one. Fleshy fruits undergo major changes in color, texture, flavor, and taste during the ripening. The color change of fruits is due to the conversion of chloroplasts to chromoplasts and the development of other pigments. The degree of firmness depends on cell wall–modifying enzymes that break the polysaccharide network to make the fruit softer (Ling et al. 2021). The breakdown of starch into sugar during ripening provides taste and flavor to fruits, making them attractive and palatable.

Fruits encounter oxidative stress during the ripening process. Methylglyoxal (MG) is one of the markers for oxidative stress and is a toxic byproduct of glycolysis and photosynthesis (Hoque et al. 2016). The mature green tomato possesses high MG levels, whereas the red ripe tomato possesses lower MG levels. A low concentration of MG can act as a signaling molecule; however, a high concentration of MG is harmful. Under stress conditions, MG accumulates in high concentrations and enhances the generation of reactive oxygen species (Hoque et al. 2012). Moreover, an elevated MG might lead to the glycation of nucleic acid and phospholipids, hindering the proper folding of proteins and nucleic acid. The role of MG has been very well explored under abiotic stress; however, the effect of different MG levels and the underlying mechanisms of those levels during fruit ripening is still elusive.

In this issue of Plant Physiology, Gambhir et al. (2023) investigated the underlying mechanism of MG detoxification through the help of glyoxalase enzymes in fruit development. To establish the role of MG in fruit ripening, they treated wild-type (WT) tomatoes with exogenous MG, which slowed down ripening compared with the control. The ripening-deficient *rin* mutants were yellow, similar to the WT tomato infiltrated with MG. These observations led to a question: What is the mechanism of lowering MG concentration during tomato fruit development? MG is a toxic chemical that is converted into a nontoxic chemical by the action of the glyoxalase enzyme (Ling et al. 2021). Therefore, the authors hypothesized that the glyoxalase system critically contributes to regulate MG levels during fruit ripening. They showed that the activities of glyoxalase enzymes increased during the ripening in WT, while these enzymatic activities were decreased in the ripening-deficient mutants. In addition, the expression analysis of the glyoxalase (GLY) family members showed that the *SlGLYI4* is the critical candidate responsible for ripening.

To validate the role of SIGY14 in fruit ripening, they showed silencing of *SlGLYI4* resulted in green tomatoes and elevated MG levels compared with red-colored control fruits, indicating that the SlGLYI14 is the primary regulator of MG detoxification during ripening. Genes related to ripening regulators were in low abundance in *SlGLYI4-*silenced fruits. One of the ripening regulators, RIN, interacts with the promoter of *SlGLYI4* and controls many ripening-related genes, suggesting that *SlGLYI4* synergistically works with RIN in tomato fruit ripening.

Color change is a typical process during fruit ripening due to enhanced activity of the carotenoid pathway (Kapoor et al. 2022). Therefore, Gambhir et al. analyzed the genes related to the carotenoid pathway in *SlGLYI4-*silenced tomato, and they showed that enhanced expression of lycopene cyclase (*LCY-B*) and *LCY-E* contributes to β-carotene accumulation and affects lycopene accumulation during ripening. The silenced fruits of *SlGLYI4* showed a decreased expression of cell wall–modifying enzymes and lower pectate lyase activity and pectin content compared with control fruit, indicating that *SlGLYI4* affects the crucial process of cell wall remodeling, which is essential for palatable fruit.

**Figure 1.**
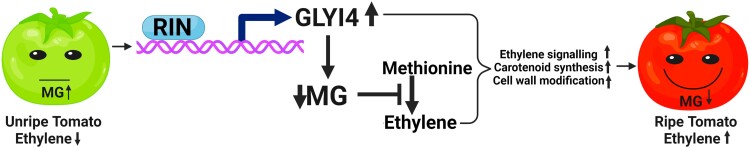
Proposed model of *SlGLYI4*-mediated detoxification of MG during fruit ripening. During fruit ripening, higher MG levels in green mature tomato decrease due to RIN-mediated activation of glyoxalase enzymes GLYI4. The elevated level of MG negatively controls the ethylene biosynthetic pathway. GLYI4 and ethylene induce the different genes related to ethylene signaling, carotenoid synthesis, and cell wall modification. The figure is adapted from Gambhir et al. 2023 and created by BioRender.com.

The phytohormone ethylene speeds ripening and affects texture and fruit quality (Liu et al. 2015). To understand the interaction of ethylene with MG, Gambhir et al. performed expression analysis of genes related to ethylene biosynthetic pathways and found that ethylene biosynthesis is compromised. They also observed lower levels of methionine, a precursor of ethylene biosynthesis, in the *SlGLYI4*-silenced fruits. Moreover, even the external methionine supply to *SlGLYI4*-silenced fruits failed to induce ethylene production, indicating that silencing of *SlGLYI4* resulted in the MG accumulation that might glycate the enzyme responsible for catalyzing the ethylene biosynthesis pathway. Collectively, the study sheds light on the regulatory mechanism of *SlGLYI4*-mediated detoxification of MG during fruit ripening that would help in manipulating traits contributing to fruit quality and shelf life (Figure 1.).
